# Interleukin-17D and Nrf2 mediate initial innate immune cell recruitment and restrict MCMV infection

**DOI:** 10.1038/s41598-018-32011-2

**Published:** 2018-09-12

**Authors:** Ruth Seelige, Robert Saddawi-Konefka, Nicholas M. Adams, Gaëlle Picarda, Joseph C. Sun, Chris A. Benedict, Jack D. Bui

**Affiliations:** 1 Department of Pathology, University of California, San Diego, CA 92093 USA; 2 Immunology Program, Memorial Sloan Kettering Cancer Center, New York, NY 10065 USA; 3 Division of Immune Regulation, La Jolla Institute for Allergy and Immunology, La Jolla, CA 92037 USA; 4 Department of Immunology and Microbial Pathogenesis, Weill Cornell Medical College, New York, NY 10065 USA; 5 Center for Infectious Disease, La Jolla Institute for Allergy and Immunology, La Jolla, CA 92037 USA

## Abstract

Innate immune cells quickly infiltrate the site of pathogen entry and not only stave off infection but also initiate antigen presentation and promote adaptive immunity. The recruitment of innate leukocytes has been well studied in the context of extracellular bacterial and fungal infection but less during viral infections. We have recently shown that the understudied cytokine Interleukin (IL)-17D can mediate neutrophil, natural killer (NK) cell and monocyte infiltration in sterile inflammation and cancer. Herein, we show that early immune cell accumulation at the peritoneal site of infection by mouse cytomegalovirus (MCMV) is mediated by IL-17D. Mice deficient in IL-17D or the transcription factor Nuclear factor (erythroid-derived 2)-like 2 (Nrf2), an inducer of IL-17D, featured an early decreased number of innate immune cells at the point of viral entry and were more susceptible to MCMV infection. Interestingly, we were able to artificially induce innate leukocyte infiltration by applying the Nrf2 activator *tert*-butylhydroquinone (tBHQ), which rendered mice less susceptible to MCMV infection. Our results implicate the Nrf2/IL-17D axis as a sensor of viral infection and suggest therapeutic benefit in boosting this pathway to promote innate antiviral responses.

## Introduction

Cytomegaloviruses (CMVs) are herpesviruses that can cause fatal disease in their respective hosts. Infections of immune- suppressed patients with human cytomegalovirus (HCMV) still cause significant complications after transplantation, and congenital infection is the leading infectious cause of brain damage and sensorineural deafness in newborns^1^. Due to its structural and biological similarity to HCMV and its host specificity, mouse cytomegalovirus (MCMV) can be safely studied in mice to gain valuable *in vivo* insights about the mechanisms of CMV pathogenesis.

Immune responses to MCMV are well described and involve both early innate as well as later adaptive immunity. Indeed, roles for natural killer (NK) cells^2^, CD8^+^ T cells^3^, CD4^+^ T cells^4^, dendritic cells (DCs)^5^, monocytes/macrophages^6^ and neutrophils^7^ have been described for the resolution of MCMV infection (reviewed in^8^). A major role for controlling MCMV infection is attributed to a subtype of NK cells expressing the activating receptor Ly49H in C57BL/6 but not BALB/C mice^9^.

Although some of the anti-pathogenic functions of different immune subsets during MCMV infection are well described, less is known about their recruitment. It is established that infiltration of leukocytes to local sites of pathogen entry involves cytokine and chemokine production by resident or early-recruited cells. Chemokines shown to be induced after MCMV infection include the neutrophil-attractant macrophage inflammatory protein (MIP)-1α^10^, the T cell-attractants CXCL9^11^ and CXCL10^11,12^ and the monocyte-, memory T cell-, neutrophil- and NK cell-attractant CCL2^13,14^. CCL2 has been established as a central mediator for recruiting macrophages and NK cells to MCMV-infected sites^14^.

Our group has recently established a role for the cytokine Interleukin (IL)- 17D during cancer progression and sterile inflammation^15,16^. IL-17D is an understudied member of the IL-17 family of cytokines, which has known functions in antipathogenic responses and leukocyte infiltration (reviewed in^17^). Interestingly, we found that IL-17D induced the chemokine CCL2, leading to the recruitment of NK cells^16^. We further showed that IL-17D expression was regulated by the transcription factor nuclear factor (erythroid-derived 2)-like 2 (Nrf2), a known sensor of oxidative stress. Notably, activating Nrf2 with the agonist *tert*-butylhydroquinone (tBHQ) induced IL-17D *in vitro* and *in vivo* and led to NK cell-mediated tumor rejection *in vivo*^15^.

Immune responses to cancer and viral infections feature several similarities, as both involve NK cells, induction of similar cytokines and chemokines, and local recruitment of immune effector cells. We have previously shown that IL-17D-deficient mice displayed sub-optimal responses to infection by MCMV and local vaccinia virus (VV) scarification^15^. By using MCMV as a model system, we now analyzed the function of IL-17D and its regulator Nrf2 with specific regard to the immune response at the peritoneal site of virus entry.

## Results and Discussion

### IL-17D deficient mice are more susceptible to MCMV infection

**Figure 1:**
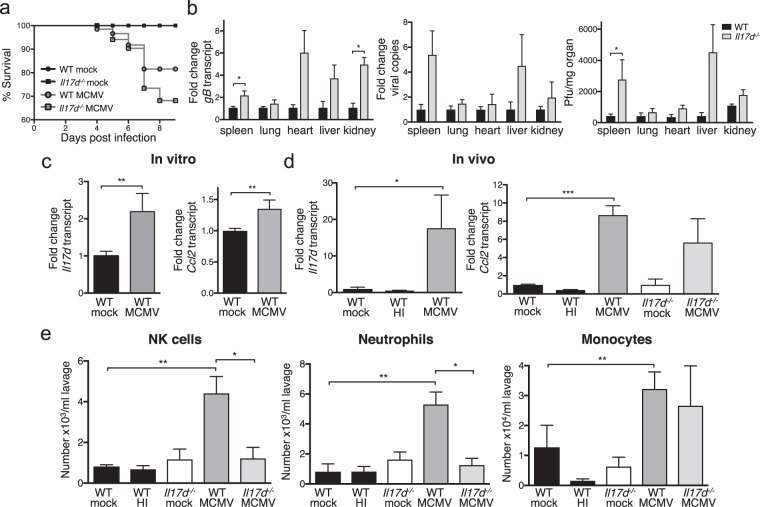
*Il17d*^*−/−*^ mice are more susceptible to MCMV infection and feature reduced immune cell recruitment into infected peritoneum. (**a**) Survival of mock- and MCMV-infected WT and *Il17D*^*−/−*^ mice. (**b**) Viral burden 5 days after infection was determined by qPCR of transcript of the viral gene *gB* (left), qPCR of DNA of the viral gene *IE1* (middle) and viral plaque assays (right). *gB* gene expression is expressed as fold change relative to expression in MCMV-infected WT mice for each organ. The amount of viral copies is expressed as fold change compared to MCMV-infected WT mice for each organ. Viral plaques are expressed as plaque-forming units (pfu)/mg organ. (**c**), (**d**) Expression of *Il17d* and *Ccl2* determined by qPCR 24 h after MCMV infection of peritoneal cells *in vitro* (**c**) or *in vivo* (**d**). Gene expression is expressed as fold change relative to gene expression in mock-infected cells (**c**) or mice (**d**). (**e**) Total numbers of NK cells (7AAD^−^/CD45^+^/CD3^*−*^/NK1.1^+^), neutrophils (7AAD^*−*^/CD45^+^/CD11b^+^/Ly6C^+^/Ly6G^high^) and monocytes/macrophage precursors (7AAD^*−*^/CD45^+^/CD11b^+^/Ly6C^+^/Ly6G^low^) in the peritoneal lavage of mock, heat- inactivated (HI) or live MCMV i.p. infected mice after 24 h. (**a**,**b**), left) Representative of three independent experiments with n = 5 or 6 mice per group. (**b**), middle) Combination of two independent experiments with n = 5 mice per group. (**b**), right) Representative of two independent experiments with n = 4 or 5 mice per group. (**c**) Combination of three independent experiments with n = 3-5 replicates per group. (**d**,**e**) Representative of three independent experiments with n = 5 mice in each group. Data are represented as mean ± SEM.

We previously showed that mice lacking IL-17D are mildly more susceptible to MCMV infection^15^, as measured by weight loss. Corroborating these findings, *Il17d*^*−/−*^ mice also featured a slightly worsened survival rate (Fig. 1a, p = 0.3) and a higher viral burden (Fig. 1b). We assessed viral burden using three different methods: 1) qPCR of the viral transcript *Glycoprotein B* (*gB*) of cDNA converted from total mRNA (Fig. 1b left, expressed as fold change compared to WT mice); 2) qPCR of the viral gene *Immediate early 1* (*IE1*) performed on total cellular DNA with viral copy number calculated from a standard curve as read-out (Fig. 1b, middle, expressed as fold change compared to WT mice) and 3) viral plaque assays (Fig. 1b right, expressed as absolute plaque forming units (pfu)/mg organ). Both qPCR methods have been shown to be directly and quantitatively correlated to viral plaque assay measurements^18,19^, and this was reproduced in our hands. Because we found the data from qPCR of converted mRNA most reproducible, least error-prone and least susceptible to bias and because we were most interested in finding *relative* differences between transcribed virus gene in WT and *Il17d*^*−/−*^ mice, we used this method for all subsequent analyses of viral burden. Corroborating our findings that *Il-17d*^*−/−*^ mice feature a mildly more severe phenotype than WT after MCMV infection, viral burdens were significantly increased in some but not all tested organs. For all experiments shown, we used mice on a C57BL/6 background.

### MCMV infection induces *Il17d* and *Ccl2* expression within 24 hours at the site of infection

We previously found that MCMV infection induces *Il17d* expression in primary murine adult fibroblasts^15^ and therefore wanted to show in our i.p. infection model if peritoneal cells could express *Il17d* in response to MCMV infection. We first lavaged peritoneal cells from uninfected mice and exposed them to MCMV *in vitro*. We found that *Il17d* was significantly upregulated in MCMV-infected cells after 24 h of *in vitro* infection, compared to mock-infected cells (Fig. 1c). This upregulation correlated with the induction of *Ccl2*, a gene we showed is a target for IL-17D in tumor models^16^. To investigate whether *Il17d* is locally induced at the point of MCMV entry, we peritoneally infected mice with MCMV *in vivo* and analyzed the lavage after 24 h. Expression of *Il17d* and C*cl2* transcript (Fig. 1d) as well as CCL2 protein (Suppl Fig. S1b) was locally increased in the peritoneal lavage from MCMV-injected compared to mock-injected mice. IL-17D protein in the peritoneal lavage and supernatant from infected cells was below the detection limit of the ELISA (not shown). There was no difference between mock-infected or heat- inactivated (HI) MCMV-infected mice, suggesting that the observed induction was indeed live virus-induced. The MCMV-mediated expression of *Ccl2* might depend on signals additional to IL-17D because *Ccl2* was still upregulated in *Il17d*^*−/−*^ animals, although not significantly. Because viral stocks of MCMV derived from infected salivary glands (SG- derived) might also contain cytokines that could, in turn, induce virus- independent cytokine activation, we also infected mice with MCMV produced and purified from cultured fibroblasts (tissue culture (TC)- derived). TC-derived MCMV also induced *Il17d* and *Ccl2* transcript and CCL2 protein (Suppl Fig. S1a), indicating that the observed inductions were directly virus-mediated.

It is not obvious which cells express IL-17D in our chosen model. We and others have shown that it is expressed in non-immune cells under baseline and cancer-bearing conditions^16,20^. Since the major resident cell populations in the peritoneum are macrophages and B cells, comprising up to 90% of the cavity^21^ (Suppl Figs S3, S6), it is likely that these are the cells that induce IL-17D in this compartment. Macrophages and B cells are immune cells but might have homeostatic, and not effector, functions in the peritoneum due to their resident character. IL-17D could also be expressed in other resident or even newly-recruited cell types.

### MCMV infection recruits innate immune cells into the peritoneum dependent on IL-17D

We next performed a detailed analysis of the major immune cell subtypes early in the peritoneum and later in the distal organs after MCMV i.p. infection in WT and *Il17d*^*−/−*^ animals (gating strategy in Suppl Fig. S2). We found that early after infection (24 h), the only effector immune cells significantly differing between mock- and MCMV-infected mice were the innate subtypes (NK cells, neutrophils, monocytes; Fig. 1e), while the numbers of T cells (CD4^+^ and CD8^+^ T cells) were similar (not shown). Immune cell numbers did not differ between mock-infected and HI MCMV-infected mice, and similar numbers of innate immune cells were again also recruited by TC-derived virus (Suppl Fig. S1c). Interestingly, the numbers of NK cells, neutrophils and monocytes were decreased in *Il17d*^*−/−*^ mice infected with MCMV. The results for monocytes are not significant, in line with our published data that recombinant IL-17D only moderately recruits monocytes^16^. We have previously immune-phenotyped *Il17d*^*−/−*^ mice and did not observe baseline differences in immune cell populations compared to WT mice in bone marrow, lymph nodes and spleen^15^. We extended our analysis to peritoneum, spleen and lung and did not see differences in innate or adaptive immune cell numbers or maturation status of NK cells (Suppl Fig. S3). Thus, the differences in recruited cell numbers after MCMV infection are not due to baseline differences in immune cell composition or NK cell maturation of *Il17d*^*−/−*^ mice.

It has been shown that the clearance of MCMV in C57BL/6 mice depends on a specific subtype of NK cells expressing the surface activating receptor Ly49H^9^. Thus, we analyzed whether MCMV i.p. infection preferably recruited this NK cell subtype early in WT mice, which might explain the increased susceptibility of *Il17d*^*−/−*^ mice to MCMV infection. We did not find a difference in Ly49H^+^ NK cell numbers or activation status of all NK cells (reflected by the expression of CD69) between mock- and MCMV-infected or between WT and *Il17d*^*−/−*^ mice at 24 h (Suppl Fig. S4a). We conclude that at this early timepoint, IL-17D does not preferentially recruit an activated subset of NK cells, but rather an increased number of NK and other innate immune cells.

### Infiltration of immune cells into distal organs is not dependent on IL-17D during MCMV infection

Since MCMV i.p. infection also leads to systemic disease, we next tested the role of IL-17D in inflammatory responses to MCMV in peripheral organs. In contrast to the induction of *Il17d* in the peritoneum at 24 h, we did not observe an upregulation in the tested organs spleen, lung, heart, liver and kidney after 36 hours (not shown), three days (not shown) or five days (Suppl Fig. S5a) of MCMV infection. Based on these results, we considered it unlikely that there is an IL-17D-mediated difference in immune cell recruitment, but still analyzed it. We chose the organs spleen and lung because these are highly infected in the MCMV model^22^ and because we wanted to include one organ with low baseline IL-17D expression (spleen;^20^ and unpublished observations) and one with high baseline IL-17D expression (lung;^20^ and unpublished observations). Similar to MCMV infection after 24 h, neutrophils and monocytes were recruited to spleen and lung five days after infection in WT mice (Suppl Fig. S5b,c). In contrast to early MCMV infection, adaptive CD8^+^ T cells were now also recruited to the lung (Suppl Fig. S5d). Moreover, we observed an increase in Ly49H^+^ NK cells and CD69 expression on NK cells after MCMV infection (Suppl Fig. S4b,c). However, as expected from the results in Suppl Fig. S5a, there was no difference in immune cell recruitment or NK cell activation in MCMV-infected *Il17d*^*−/−*^ compared to WT mice (Suppl Figs S4b,c, S5b–d). We conclude that early innate immune cell recruitment to peritoneal MCMV infection is mediated by IL-17D, while the recruitment of immune cells to systemically infected organs at later time points depends on mechanisms distinct from IL-17D. This might explain why the systemic infection severity of *Il17d*^*−/−*^ mice is only slightly increased compared to WT mice (Fig. 1a,b). Additionally, we cannot exclude that the observed effect of IL-17D on immune cell recruitment is peritoneum-specific.

### IL-17D expression is beneficial during MCMV infection in the absence of adaptive immunity

**Figure 2:**
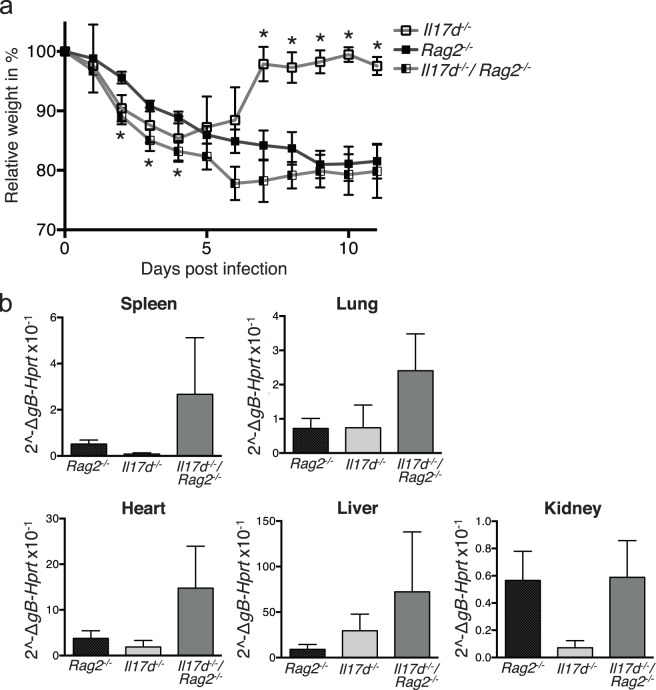
*Il17d*/*Rag2* double deficient mice are more susceptible to MCMV infection than *Il17d* or *Rag2* single deficient mice. (**a**) Weight of MCMV-infected *Il17d*^*−/−*^, *Rag2*^*−/−*^ and *Il17d*^*−/−*^*/ Rag2*^*−/−*^ mice. (**b**) Viral burden as determined by qPCR of transcript of the viral gene *gB* in spleen, lung, heart, liver and kidney 5 days after infection, expressed relative to housekeeping gene. Data are combination of two independent experiments with n = 5 mice per group. Data are represented as mean ± SEM. Stars in (**a**) represent significant differences between *Rag2*^*−/−*^ and *Il17d*^*−/−*^*/Rag2*^*−/−*^ (days 2–4) and between *Il17d*^*−/−*^ and *Il17d*^*−/−*^*/Rag2*^*−/−*^ mice (days 7–11).

To examine the role of IL-17D in the absence of adaptive immunity, we crossed the *Il17d*^*−/−*^ mice to *Rag2*^*−/−*^ mice that lack adaptive immunity^23^ and infected double deficient progeny with MCMV. Similar to what has been published for *Rag1*^*−/−*^ mice^24^, *Rag2*^*−/−*^ mice were not able to recover from MCMV infection (Fig. 2a). *Il17d/Rag2* double deficient mice were more susceptible to MCMV infection than *Il17d* or *Rag2* single deficient mice, reflected by an earlier increase in weight loss, a decreased recovery (Fig. 2a) and a higher viral burden in most infected organs (Fig. 2b). We conclude that the early local recruitment of innate immune cells by IL-17D is enough to render mice slightly more resistant to MCMV infection, but that adaptive immunity is required for efficient systemic MCMV clearance at later time points.

### Nrf2- deficient mice are more susceptible to MCMV infection

**Figure 3:**
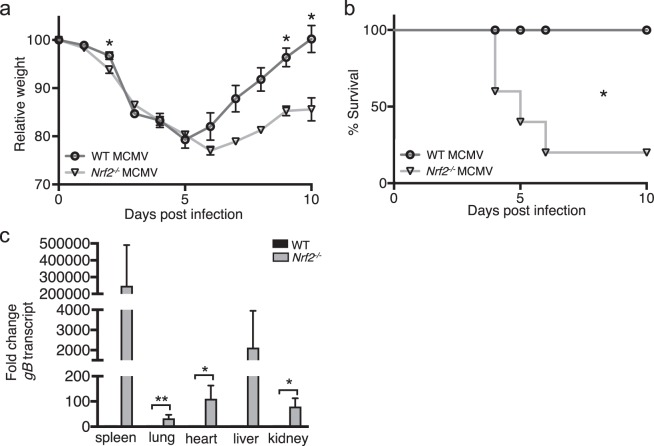
*Nrf2*^*−/−*^ mice are more susceptible to MCMV infection.Weight (**a**) and survival (**b**) of MCMV-infected WT and *Nrf2*^*−/−*^ mice. (**c**) Viral burden as determined by qPCR of transcript of the viral gene *gB* in spleen, lung, heart, liver and kidney 5 days after infection. *gB* transcript expression is expressed as fold change relative to expression in MCMV-infected WT mice for each organ.Representative of two independent experiments with n = 5 or 6 mice per group. Data are represented as mean ± SEM.

We have previously shown that IL-17D expression is regulated by the transcription factor Nrf2 in cancer cells^15^. We now sought to explore the role of Nrf2 during MCMV infection and whether it plays a role for IL-17D induction. We therefore infected *Nrf2*^*−/−*^ mice with MCMV. As hypothesized, *Nrf2*^*−/−*^ mice were highly susceptible to MCMV infection as observed by a 23% decrease in weight, a worsened ability to recover (Fig. 3a), a significantly decreased survival rate (Fig. 3b), and an increase in viral burden of infected organs (Fig. 3c). Interestingly, the phenotype was more profound than that of *Il17d*^*−/−*^ mice, suggesting that Nrf2 likely regulates other mediators of resistance to virus infection.

### MCMV-induced *Il17d* expression depends on Nrf2

**Figure 4:**
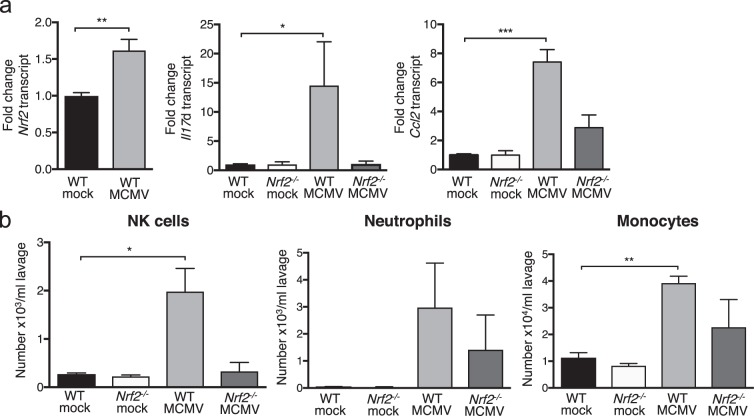
MCMV infection induces *Nrf2*; and the recruitment of innate immune cells to the site of MCMV entry is reduced in *Nrf2*^*−/−*^ mice. (**a**) Expression of *Nrf2* (left), *Il17d* (middle) and *Ccl2* (right) determined by qPCR 24 h after i.p. MCMV infection in WT and *Nrf2*^*−/−*^ mice. Gene expression is expressed as fold change relative to expression in mock-infected mice. Combination of two independent experiments with n = 3 or 4 mice per group. (**b**) Total numbers of NK cells (7AAD^*−*^/CD45^+^/CD3^*−*^/NK1.1^+^), neutrophils (7AAD^*−*^/CD45^+^/CD11b^+^/Ly6C^+^/Ly6G^high^) and monocytes/macrophage precursors (7AAD^*−*^/CD45^+^/CD11b^+^/Ly6C^+^/Ly6G^low^) in the peritoneal lavage of mock or MCMV i.p. infected mice after 24 h. Representative of two independent experiments with n = 5 mice in each group.Data are represented as mean ± SEM.

Since MCMV infection induced *Il17d* (Fig. 1c,d) and we have shown that Nrf2 regulates *Il17d* in cancer^15^, we next analyzed whether Nrf2 was induced after MCMV infection and whether *Il17d* expression depended on Nrf2. Indeed, we found *Nrf2* to be upregulated 24 h after *in vivo* i.p. infection (Fig. 4a). Like shown in Fig. 1d, MCMV infection also induced *Il17d* and *Ccl2* but *Il17d* upregulation was completely abolished in *Nrf2*^*−/−*^ mice, indicating that MCMV-induced *Il17d* expression requires Nrf2. Similar to *Il17d*^*−/−*^ mice (Fig. 1d), *Ccl2* induction was modestly, but not significantly, induced in *Nrf2*^*−/−*^ mice, suggesting that additional mechanisms control virus-induced *Ccl2* expression.

### MCMV infection recruits innate immune cells into the peritoneum dependent on Nrf2

We next examined immune cell recruitment 24 h after peritoneal MCMV infection in WT and *Nrf2*^*−/−*^ animals. As already shown in Fig. 1e, MCMV infection increased the number of innate immune cells (NK cells, neutrophils, monocytes) compared to mock infection. Notably, *Nrf2* deficiency reduced the numbers of these cells recruited to the MCMV-infected peritoneum, similar to *Il17d* deficiency (Fig. 4b).

To exclude that *Nrf2*^*−/−*^ mice have baseline differences in the composition of their immune cell subtypes, we immune-phenotyped the peritoneum, spleen and lung of these mice (Suppl Fig. S6). To our knowledge, this has not been done before although anemia has been reported in *Nrf2*^*−/−*^ mice^25^. The only significant differences observed were a decrease in macrophages in the peritoneum as well as a decrease of neutrophils in the lungs of *Nrf2*^*−/−*^ mice. We conclude that *Nrf2*^*−/−*^ mice have a slightly different composition of their peritoneum and lung-resident immune cells. We cannot exlude that these differences influence immune responses during MCMV infection. Further research is needed to clarify whether the reduced percentage of peritoneal macrophages in *Nrf2*^*−/−*^ mice affects the recruitment of immune cells into this compartment due to induction of other cytokines. However, we consider it unlikely that it affects the IL-17D-mediated recruitment since IL-17D expression is absent in peritoneal cells from *Nrf2*^*−/−*^ mice (Fig. 4a).

### tBHQ protects from MCMV infection

**Figure 5:**
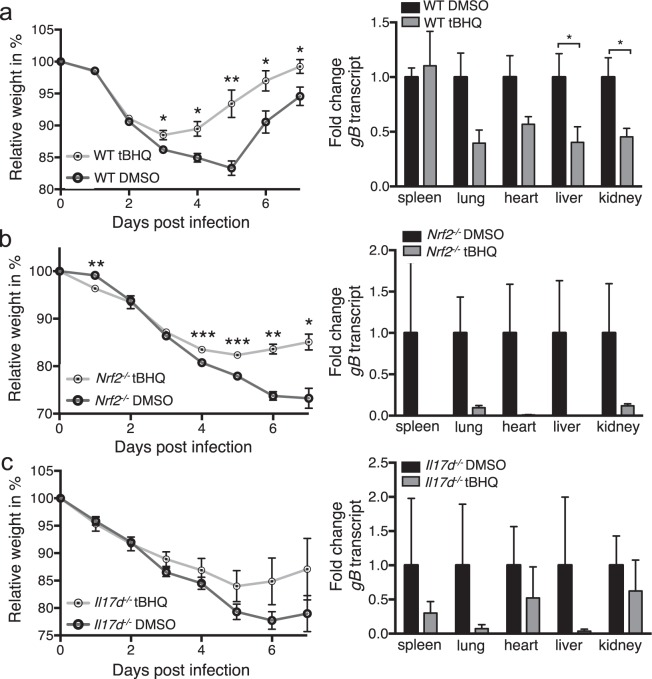
tBHQ injection decreases susceptibility to MCMV.WT (**a**), *Nrf2*^*−/−*^ (**b**) or *Il17d*^*−/−*^ (**c**) mice were i.p. infected with MCMV and injected with 50 mM tBHQ/DMSO (or PBS/DMSO), and monitored daily for weight loss (left). Viral burden was determined by qPCR of the viral gene *gB* in spleen, lung, heart, liver and kidney (right) and expressed as fold change relative to expression in DMSO-injected mice.Representative of three (**a**,**c**) or two (**b**) independent experiments with n = 5 mice per group. Data are represented as mean ± SEM.

We have recently published that topical application of the Nrf2 activator tBHQ on tumors induces IL-17D, which leads to NK cell recruitment and subsequent tumor rejection^15^. We next investigated if tBHQ could have a similar protective effect during viral infection. We found that injection of tBHQ protected WT mice from MCMV infection, resulting in significantly less weight loss and lower viral burden in most infected organs (Fig. 5a). Surprisingly, MCMV-infected *Nrf2*^*−/−*^ mice treated with tBHQ were significantly less susceptible to infection than their DMSO-injected counterparts (Fig. 5b). However, tBHQ was not able to rescue from MCMV infection to a level as observed in WT mice. This suggests that tBHQ activates other protective pathways independent of Nrf2. We obtained similar results in *Il17d*^*−/−*^ mice treated with tBHQ in that tBHQ still partly rescued the MCMV-induced weight loss and viral burden (Fig. 5c), although not significantly and not to WT levels.

### tBHQ locally induces Nrf2 activity, *Il17d* and *Ccl2* expression and recruits innate immune cells

**Figure 6:**
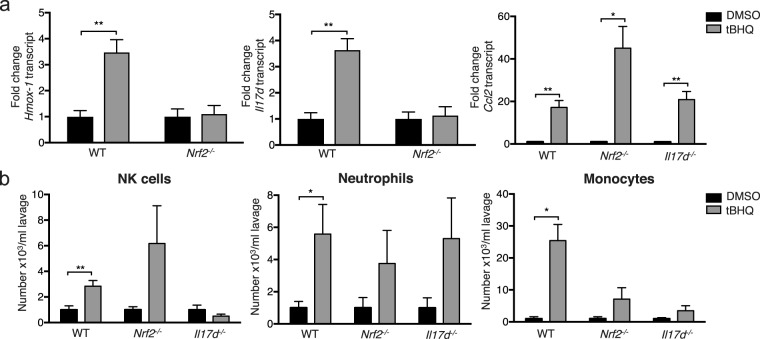
tBHQ injection induces *Hmox-1*, *Il17d* and *Ccl2* expression and the recruitment of innate immune cells. (**a**) Expression of Nrf2 target gene *Hmox-1* (left), *Il17d* (middle) and *Ccl2* (right) determined by qPCR 24 h after i.p. injection of 50 mM tBHQ/DMSO (or PBS/DMSO) in WT, *Nrf2*^*−/−*^ or *Il17d*^*−/−*^ mice. Gene expression is expressed as fold change relative to expression in DMSO-injected mice. Combination of three (two for *Nrf2*^*−/−*^) independent experiments with n = 5 or 6 (n = 3 or 4 for *Nrf2*^*−/−*^) mice per group. (**b**) Numbers of NK cells (7AAD^*−*^/CD45^+^/CD3^*−*^/NK1.1^+^) (left), neutrophils (7AAD^*−*^ /CD45^+^/ CD11b^+^/ Ly6C^+^/ Ly6G^high^) (middle) and monocytes/macrophage precursors (7AAD^*−*^/CD45^+^/CD11b^+^/Ly6C^+^/Ly6G^low^) (right) in the peritoneal lavage of PBS/DMSO or tBHQ/DMSO i.p. injected WT, *Nrf2*^*−/−*^ or *Il17d*^*−/−*^ mice after 24 h. Representative of three (two for *Nrf2*^*−/−*^) independent experiments with n = 5 or 6 (n = 3 or 4 for *Nrf2*^*−/−*^) mice per group.Data are represented as mean ± SEM.

We sought to further analyze the mechanism of tBHQ-mediated protection against MCMV infection. We first tested whether Nrf2 is activated by tBHQ in this particular model of i.p. injection and whether it induces IL-17D. Indeed, we observed an upregulation of the Nrf2 target gene *Hmox-1* in WT, but not *Nrf2*^*−/−*^ mice (Fig. 6a). Moreover, tBHQ treatment induced *Il17d* in WT, but not *Nrf2*^*−/−*^ mice, showing again that IL-17D expression is regulated by Nrf2. The immune cell-recruiting chemokine *Ccl2* was also highly upregulated by tBHQ injection. In contrast to *Hmox-1* and *Il17d*, however, *Ccl2* was still induced in both *Nrf2*^*−/−*^ and *Il17d*^*−/−*^ animals, suggesting that tBHQ activates other pathways apart from Nrf2 and IL-17D. This is in contrast to its application in cancer where we have shown that it is not effective if Nrf2 or IL-17D are missing^15^. We next analyzed immune cell recruitment into the peritoneum after local tBHQ injection. Notably, tBHQ mobilized innate immune cell infiltration after 24 h (Fig. 6b). Interestingly, these cells were still recruited in *Nrf2*^*−/−*^ mice, although not significantly and, in the case of monocytes, less profoundly. Likewise, neutrophils and monocytes still infiltrated in high numbers into the tBHQ-injected peritoneum even in the absence of IL-17D. Similar to the absence of Nrf2, the increase in number was not significant for neutrophils and monocytes and much lower for monocytes. NK cells were no longer recruited after tBHQ injection into the peritoneum of *Il17d*^*−/−*^ mice. This shows that although other mechanisms apart from Nrf2 and IL-17D regulate neutrophil and, in part, monocyte recruitment after tBHQ injection, the recruitment of NK cells solely depends on IL-17D. We conclude that other mechanisms besides Nrf2 and IL-17D could regulate certain innate immune cell infiltration after tBHQ injection. Other studies have found that tBHQ can also activate Akt^26^ and its downstream target endothelial nitric oxide synthase (eNOS)^27^. It is possible that tBHQ acts through one of these or another yet unknown pathway in our system. It remains open how NK cell recruitment in *Il17d*^*−/−*^ mice is not induced although *Ccl2* is still upregulated. We speculate that IL-17D induces other NK cell-recruiting chemokines after tBHQ activation. Either way, tBHQ bears potential to serve as immune therapy or prophylaxis not only for cancer^15^, but also for viral infection.

### Poly (dA:dT) induces *Il17d* dependent on Nrf2

**Figure 7:**
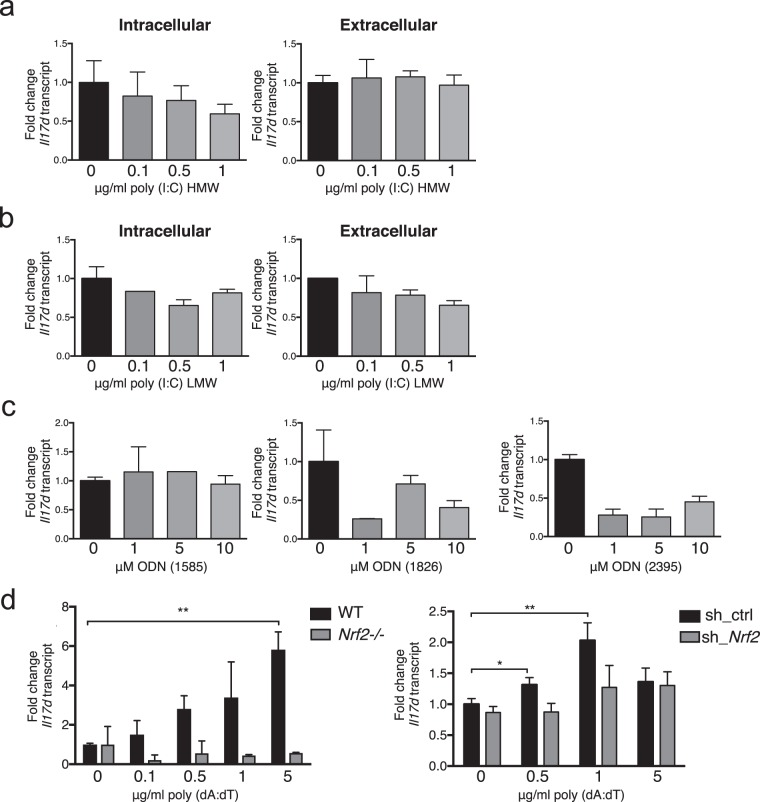
Poly (dA:dT), but not poly (I:C) or ODNs, induces *Il17d* expression dependent on Nrf2. (**a–c**) Expression of *Il17d* in freshly isolated peritoneal cells determined by qPCR 24 h after intracellular (left) or extracellular (right) stimulation with poly (I:C) HMW (**a**), LMW (**b**) or different kinds of ODNs (**c**). Gene expression is expressed as fold change relative to expression in vehicle stimulated controls. (**d**) Expression of *Il17d* in freshly isolated WT or *Nrf2*^*−/−*^ peritoneal cells (left) or B16 melanoma cells bearing a stable *Nrf2* knockdown (right) determined by qPCR 24 h after stimulation with poly (dA:dT).Gene expression is expressed as fold change relative to expression in vehicle stimulated or sh_ctrl cells.Representative of three (two for *Nrf2*^*−/−*^) independent experiments with n = 3 replicates per group. Data are represented as mean ± SEM. HMW = high molecular weight, LMW = low molecular weight, ODN = oligodeoxynucleotide.

Infections are usually sensed by Toll-like receptors (TLRs) that bind to extracellular or intracellular pathogen-associated molecular patterns (PAMPs). For MCMV, previous studies have found a role for TLR3 and TLR9 in mediating responses^28^. Therefore, we examined whether agonists for TLR3 or TLR9 were sufficient to induce *Il17d* in uninfected cells. We did not observe an upregulation of *Il17d* expression after stimulation with the TLR3 ligand poly inosine-cytidylic acid (I:C) or the TLR9 ligand CpG oligodeoxynucleotides (ODN) (Fig. 7a–c). We also did not detect induction of *Il17d* by intracellular delivery of poly (I:C), which is sensed by the pattern recognition receptor Retinoic acid-inducible gene (RIG)-I/ Melanoma- differentiation- associated gene (MDA)-5^29^ (Fig. 7a,b). We next investigated whether the MCMV-induced upregulation of *Il17d* might go through cytosolic DNA sensors such as the Absent in melanoma (AIM)2 inflammasome, recently shown to be important for MCMV infection^30^, or the cyclic GMP-AMP synthase (cGAS)/Stimulator of Interferon genes (STING) pathway, recently shown to be important for HCMV infection^31^. Indeed, the stimulator of these pathways poly deoxyadenylic-deoxythymidylic (dA:dT) did induce *Il17d* in *in vitro* stimulated peritoneal cells (Fig. 7d). This induction was prevented in *Nrf2*^*−/−*^ mice. To confirm these data in a different *in vitro* system, we made use of previously in-house created B16 melanoma cell lines that feature an shRNA-mediated stable knockdown of *Nrf2* expression^15^. In B16 sh_ctrl cell lines, *Il17d* was induced with different concentrations of poly (dA:dT), which was prevented when *Nrf2* was knocked down (Fig. 7d). To sum up, *Il17d* was induced by poly (dA:dT) stimulation and this depended on Nrf2 in different kinds of cells. We suggest that the AIM2 inflammasome or the cGAS/STING pathway might be the MCMV-sensing complexes inducing the Nrf2/IL-17D pathway in our system. Nrf2 has already been shown to be required for AIM2 inflammasome activation^32^. More research is needed to clarify the role of inflammasomes for IL-17D biology.

To sum up, we here document a role for IL-17D and Nrf2 in effective immune cell recruitment to peritoneal viral infection. We have previously established an important function for IL-17D and its regulator Nrf2 during cancer immune surveillance and even proposed the Nrf2/IL-17D/innate immunity pathway as a target for cancer immune therapy^15^. Here, we show in more detail that the same pathway is also involved in optimal immunity against viral infection. Mice deficient in IL-17D or Nrf2 featured higher susceptibility to MCMV infection likely caused by a defective ability to recruit innate immune cells to the site of viral entry. Although MCMV has been shown to be able to travel freely after i.p. infection^22^, we suggest that an innate peritoneal immune response could limit infection by eliminating free virus or lysing the cells that are peritoneally infected.

The ancient family of IL-17 cytokines has been described as important for anti-pathogen defense^17,33,34^. The most studied members IL-17A and F, which are expressed by Th17 cells, have been implicated in host immune responses against bacteria and fungi by recruitment of neutrophils^16,35–37^. IL-17C is expressed by epithelial cells and activates the autocrine production of antibacterial peptides and pro-inflammatory molecules, mediating immunity to extracellular pathogens, similar to IL-17A^38,39^. The roles for IL-17B and E are less clear but IL-17E has been studied in anti-helminth immunity and recruitment of eosinophils^40^. Less is known about IL-17 involvement in virus infections. Both pro- and anti-viral roles for IL-17A/F have been suggested for a number of viruses, including VV^41,42^, influenza^43,44^, Hepatitis B virus (HBV) (reviewed in^45,46^), Hepatitis C virus (HCV) (reviewed in^47^), Dengue virus (reviewed in^48^), respiratory syncytial virus (RSV) (reviewed in^49^), and Theiler’s murine encephalomyelitis virus (TMEV)^50–52^. Most of these publications assign the function of IL-17 to its expression in adaptive Th17 cells and subsequent induction of cytokine secretion, while only few include a leukocyte recruitment phenotype. To our knowledge, IL-17B, C or E have not been implicated in viral infections thus far. Our studies now solidify a role for IL-17D in antiviral responses. Given the ancient role of other IL-17 family members during infection and IL-17D’s function during local innate immune responses against viruses, we propose that it evolved early to mediate challenges caused by intracellular pathogens, maybe even before the evolution of adaptive immunity. Indeed, the finding that *Il17d/Rag2* double deficient mice are even more susceptible to MCMV than single deficient mice documents a role for IL-17D in the absence of adaptive immunity, perhaps as an early local sentinel of infection.

Nrf2 is known to be required for effective antitumor responses, but paradoxically has both pro- and anti-cancer functions due to its induction of a host-protective antioxidant program (reviewed in^53^). Similarly, both promoting and inhibitory roles have been reported for Nrf2 in inflammatory responses and bacterial infections^54–57^. Research on Nrf2 in virus infections is emerging, suggesting an involvement of Nrf2 in a number of *in vitro* viral infections, e.g. HBV^58^, HCV^59,60^, influenza^61^, Dengue virus^62^ and HIV^63,64^, mainly attributed to its antioxidant activities. Some papers also use *in vivo* infections of RSV^65^ and influenza^66^ to show that *Nrf2*^*−/−*^ mice are more susceptible. To our knowledge, no study has observed increased mortality of *Nrf2*^*−/−*^ mice in response to viral infection, although one study demonstrated decreased survival after influenza infection, but only after the additional stimulus of cigarette smoke^66^. No study thus far has analyzed Nrf2 during MCMV infection although one paper showed that Nrf2 was upregulated after *in vitro* HCMV infection^67^, suggesting a role for Nrf2 during CMV infections. Here, we show that *Nrf2*^*−/−*^ mice are highly susceptible to MCMV infection and we propose that this is in part due to a reduced recruitment of innate immune cells. We do not exclude that Nrf2’s antioxidant functions contribute to resistance to MCMV in our model but we suggest that the infiltration of innate leukocytes accounts for part of the phenotype. Since we have shown that Nrf2 regulates IL-17D^15^, we suggest that Nrf2 mediates the recruitment of immune cells by inducing IL-17D. However, additional mechanisms likely contribute to the reduced resistance to MCMV because *Nrf2*^*−/−*^ mice were far more susceptible to infection than *Il17d*^*−/−*^ mice. We do demonstrate, though, that IL-17D induction solely goes through Nrf2 because it was completely abrogated in *Nrf2*^*−/−*^ mice after MCMV infection.

## Conclusions

**Figure 8:**
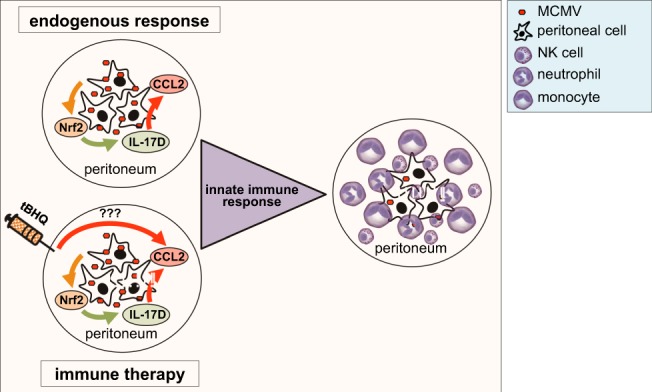
Nrf2 and IL-17D mediate recruitment of innate immune cells to the peritoneum after MCMV infection. Nrf2, IL-17D and CCL2 are locally induced after MCMV infection, resulting in innate immune cell recruitment into the peritoneum and partial viral clearance. Artificially activating Nrf2 with tBHQ has similar effects and could be used as antiviral immune therapy.

In conclusion, we present a heretofore-unknown function of Nrf2 and IL-17D during MCMV infection. Deficiency in either protein reduced early infiltration of innate leukocytes into the peritoneum (Fig. 8), leading to slightly increased disease progression. Moreover, activating Nrf2 using an agonist led to protective effects. We suggest that application of the Nrf2 activator tBHQ could serve as a viral immune therapy or prevention through IL-17D-mediated recruitment of NK and other innate immune effector cells. Researching molecular mechanisms of immune cell recruitment in the well-established experimental model of MCMV infection might also produce generalizable data that could be applied to other latent herpesvirus infections.

## Materials and Methods

All methods were performed in accordance with the relevant guidelines and regulations.

### Cell Lines

B16F10 melanoma and 3T3 cells were cultured in complete RPMI 1640 (Gibco) supplemented with 10% FCS (Atlanta Biologics). shRNA mediated knockdown of *Nrf2* in B16 cells was achieved as previously described^15^.

### Antibodies

Directly fluorophore-coupled antibodies used for flow cytometry in this work were: α-B220 (clone RA3-6B2), α-CD3 (clone 17 A.2), α-CD4 (clone GK1.5), α-CD8a (clone 53-6.7), α-CD11b (clone M1/70), α-CD45 (clone 30-F11), α-CD69 (H1.2F3), α-F4/80 (clone BM8), α-Ly6C (clone HK1.4), α-Ly6G (clone 1A8), α-Ly6H (clone 3D10), α-NK1.1 (PK136), and their respective isotype control antibodies (all from BioLegend).

### Mice

All mouse experiments were approved by the UCSD Institutional Animal Care and Use Committee (IACUC protocol #S06201) and were performed in accordance with the relevant guidelines and regulations. In-house bred mice of C57BL/6, BALB/C, *Rag2*^*tm1*.*1Cgn*^ *(Rag2*^*−/−*^*)*, *Nfe2l2*^*tm1Ywk*^/J (*Nrf2*^*−/−*^) (in-house backcrossed to C57BL/6) (all from Jackson) and *Il17d*^*tm1Lex*^/*Mmucd* (*Il17d*^*−/−*^) (in-house backcrossed to C57BL/6) (UC Davis MMRC) and *Rag2*^*−/−*^*/Il17d*^*−/−*^ double deficient mice (in-house crossed) were used in this work. All viral infection experiments shown in the figures were performed with mice on a C57BL/6 background. Salivary gland (SG) virus preparations were derived from BALB/C mice.

### Viral Preparations

MCMV Smith Strain was kindly donated by Dr. Elina Zuniga (UCSD) and viral stocks were prepared from SG homogenates of BALB/C mice intraperitoneally (i.p.) infected with 1 × 10^4^ pfu/mouse. Homogenates were prepared in sterile PBS, centrifuged at 1800g for 10 min and supernatant was used for subsequent experiments. MCMV Smith ‘tissue culture’ (TC)- derived stocks were derived from the BAC-cloned strain containing a wild-type viral MCK-2 gene^68^. The Smith BAC was first electroporated into 3T3 cells, replication was allowed to proceed until 100% cytopathogenic effect (cpe) was achieved and viral supernatants were harvested. Supernatants were then passaged 4 additional times, infecting at an MOI of ~0.1 and letting infection proceed to 100% cpe, allowing for excision of the BAC cassette from the replicating viral genome^69^. This was done 3 independent times, and comparisons of resulting virus showed similar genomic RFLP patterns, undetectable BAC-cassette sequences and similar replication levels in C57BL/6 and Balb/c mice at day 4 in spleen and liver and day 12 in salivary glands. One of these stocks was chosen as a ‘1° stock’, which was then passaged twice more in 3T3 cells to generate a ‘seed-stock’ and then a ‘working stock’, which was used for the described experiments. Viral titers were determined *in vitro* by plaque assays on 3T3 cells as described^28^. For some experiments, SG virus was heat- inactivated by incubation at 56 °C for 30 min.

### Viral Infections

*In vitro*, freshly lavaged peritoneal cells were infected with 1 × 10^5^ pfu/4 × 10^5^ cells for 2 h, washed and lysed in PureLink^TM^ RNA mini kit (Ambion) lysis buffer after 24 h.

*In vivo*, age and sex-matched eight to twelve week old mice were infected with 3 × 10^5^ pfu/mouse i.p., weighed and monitored daily for disease progression. For some experiments, mice were infected with 1 × 10^6^ pfu/mouse of TC- derived MCMV. Immune cell recruitment into the peritoneum was determined 24 h after infection by lavaging the peritoneal cavity twice with 10 ml of PBS/3 mM EDTA, counting the total number of cells in the lavage and subsequent flow cytometer analysis^70^. Spleen and lung were processed 5 days after infection by mincing and subsequent collagenase (Sigma-Aldrich) digestion (only for lung), straining through a 70 μm filter followed by erythrocyte lysis with red blood cell lysis buffer (BioLegend). Viral burden in spleen, lung, liver, heart and kidney homogenates was determined by plaque assays on 3T3 cells as described^28^ or by qPCR analysis of the MCMV gene transcript *Glycoprotein B* (*gB*), determining the expression of transcribed virus RNA relevant to housekeeping RNA, directly correlating to disease severity and plaque assays. For absolute quantification of viral DNA copies, we performed qPCR of the MCMV *Immediate early 1* (*IE1*) gene and calculated the number of target gene copies using a standard curve (see below under “Quantitative PCR”). Both qPCR methods are directly and quantitatively correlated to viral plaque assay measurements^18,19^. For some experiments, mice were i.p. injected with 50 mM tBHQ in PBS:DMSO (4:1).

### FACS analysis

Single cell suspensions from peritoneal lavage, spleen and lung were incubated with 1 μg/100 μl of αCD16/CD32 (clone 2.4G2, BD Biosciences) FC-receptor block before staining the cells with 1 μg/100 μl of the respective antibodies. Samples were analyzed on a FACS CANTO II (BD Biosciences). We defined 7-aminoactinomycin D (7AAD)^*−*^/CD45^+^ immune subtypes in the peritoneal lavage, spleen and lung as follows: CD4^+^ T cells (CD3^+^/CD4^+^), CD8^+^ T cells (CD3^+^/CD8^+^), B cells (B220^+^), macrophages (F4/80^+^), NK cells (CD3^*−*^/NK1.1^+^), neutrophils (CD11b^+^/Ly6C^+^/Ly6G^high^) and monocytes/macrophage precursors (CD11b^+^/Ly6C^+^/Ly6G^low^) (gating strategy in Suppl Fig. S2).

### Quantitative PCR

Whole tissues were either directly used or frozen at −80 °C in Trizol (Ambion) for no longer than one week. RNA was isolated according to the manufacturer’s manual without modifications. RNA from macrophages or peritoneal lavage cells was directly isolated using the PureLink^TM^ RNA mini kit (Ambion) according to the manufacturer’s manual without modifications. Concentrations and A_260/280_ absorbances were measured with NanoDrop 2000 Spectrophotometer (Thermo Scientific). Samples with A_260/280_ ~2.0 were considered pure RNA and used for cDNA generation. 2 μg of RNA was converted into cDNA with the High-Capacity cDNA Reverse Transcription Kit (Thermo Fisher Scientific, product ID 4368814) according to the manufacturer’s manual and concentrations and A_260/280_ were measured with NanoDrop, typically yielding DNA with A_260/280_ of 1.8. qPCR reactions were prepared with SYBR Green PCR Master Mix (Thermo Fisher Scientific, product ID 4309155), which includes AmpliTaq Gold DNA Polymerase, according to the manufacturer’s manual with 2 μM each of forward and reverse primers and performed on a Bio-Rad CFX96 machine. Each sample was run in triplicates and the reaction conditions were as follows: initial denaturation at 95 °C for 10 minutes followed by 95 °C for 10 seconds and 60 °C for 1 min for 40 cycles. The no template control (NTC) gave C_T_ values of >40 for each experiment. *Hprt* was used as the housekeeping reference gene because it did not produce significant variations in C_T_ values in any of the tested organs or conditions. Relative quantification of gene expression was performed using the 2^*−*ΔΔC^_T_ method^71^, where gene expression was expressed as fold change relative to gene expression of the respective control relative to housekeeping reference in each experiment. The control for each experiment is indicated in the figure legends. We used this method to determine inductions of *Il17d*, *Ccl2*, *Nrf2* and *Hmox-1* as well as to detect transcribed MCMV *gB* relative to housekeeping gene. For absolute quantification of MCMV target gene copies in infected tissues, tissue was homogenized in PBS and frozen at −80 °C for no longer than one week. DNA was isolated with the QIAamp® DNA Mini kit (Qiagen) according to the manufacturer’s manual without modifications. qPCR of the MCMV gene *IE1* was performed in triplicates as described in detail in^19,72^. This method determines absolute target gene copy numbers by relating the qPCR signal to a standard curve generated from plotting average C_T_ values against the logarithm of target template molecules obtained from a control plasmid, followed by a sum of least squares regression analysis^19^. The following primer sequences were used: *Ccl2* (fw- TAAAAACCTGGATCGGAACCAAA; rv- GCATTAGCTTCAGATTTACGGGT), *gB* (fw- GAAGATCCGCATGTCCTTCAG; rv- AATCCGTCCAACATCTTGTCG), *Hmox-1* (fw-TGAAGGAGGCCACCAAGGAGG; rv- AGAGGTCACCCAGGTAGCGGG), *Hprt* (fw- GCTTGCTGGTGAAAAGGACCTCTCGAAG; rv- CCCTGAAGTACTCATTATAGTCAAGGGCAT), *IE1* (fw- TCGCCCATCGTTTCGAGA; rv- TCTCGTAGGTCCACTGACCGA), *Il17d* (fw- AGCTTGTCCATGCTGGAGTT; rv- CTCTACGGGGAGGAGGACTT), and *Nrf2* (fw CAGTCTTCACTGCCCCTCAT; rv- TCTGTCAGTGTGGCTTCTGG). Primers for *Ccl2* (NM_011333.3,^73^), *gB* (NC_004065,^74^), *Hmox-1* (NM_010442,^75^), *Hprt* (NM_013556,^76^), *IE1* (GenBank L07320.1,^72^) and *Nrf2* (NM_010902.4,^77^) were previously used and validated in other publications and additionally BLASTed for target specificity before use. Primers for *gB* were additionally validated by giving strong signals in MCMV-infected tissues, but no signals in mock-infected tissues. Additionally, the 2^*−*ΔC^_T_ value relative to housekeeping gene directly correlated with disease severity in MCMV-infected animals. Primers for *Il17d* (NM_145837.3) were previously designed by our group^15^ and validated by producing the correctly sized product in an agarose gel, giving robust signals in tissues from WT mice, but giving no detectable signal in tissues from *Il17d*^*−/−*^ mice. Additionally, we tested that these primers give reliably high signals in IL-17D-overexpressing cells, but reliably low signals in cells known to express low levels of IL-17D^16^.

### Elisa

Enzyme-linked immunosorbent assay (ELISA) for CCL2 was performed according to the manufacturer’s manual (R&D Systems). ELISA for IL-17D was performed using the same protocol with rat-α-mIL-17D (R&D Systems, clone #312724) as capture antibody at 200 ng/ml and biotinylated goat α-mIL-17D (R&D Systems, immunogen accession #Q8K4C4) as detection antibody at 50 ng/ml.

### Poly (I:C), poly (dA:dT) and oligodeoxynucleotide stimulations

Freshly lavaged peritoneal cells were stimulated with naked poly (I:C) or ODNs 1585, 1826, or 2395 (all invivogen) for 24 h and lysed in RNA lysing buffer (Ambion). For intracellular stimulation, cells were transfected with complexes between poly (I:C) or poly (dA:dT) (invivogen) and the transfection reagent LyoVec^TM^ according to the manufacturer’s manual.

### Statistical Analysis

Statistical significance was determined by the Mann-Whitney test with the Prism 6.0 software (GraphPad Software, Inc.). For survival curves, statistical significance was determined by the Log-rank (Mantel-Cox) test. Error bars are depicted using SEM. All experiments were repeated at least twice. *P < 0.05, **P < 0.01, ***P < 0.001, or no asterisks for not significant, in all data shown.

## Electronic supplementary material

Supplementary Figures

## Data Availability

All data generated or analyzed during this study are included in this published article (and its Supplementary Information files). If additional details are desired, they are available from the corresponding author on request.
