# Supplementary material for: Interleukin-17D and Nrf2 mediate initial innate immune cell recruitment and restrict MCMV infection
Source: Sci Rep. 2018 Sep 12;8:13670. doi: 10.1038/s41598-018-32011-2 (PMC6135835; doi:10.1038/s41598-018-32011-2)
Supplement: Supplementary file 1 — Supplementary Figures [file 41598_2018_32011_MOESM1_ESM.pdf]

Interleukin-17D and Nrf2 mediate initial innate immune cell recruitment and restrict MCMV infection

Ruth Seelige, Robert Saddawi-Konefka, Nicholas M. Adams, Gaëlle Picarda, Joseph C. Sun, Chris A. Benedict, and Jack D. Bui

Supplementary Figure S1. Effects of TC-derived MCMV.

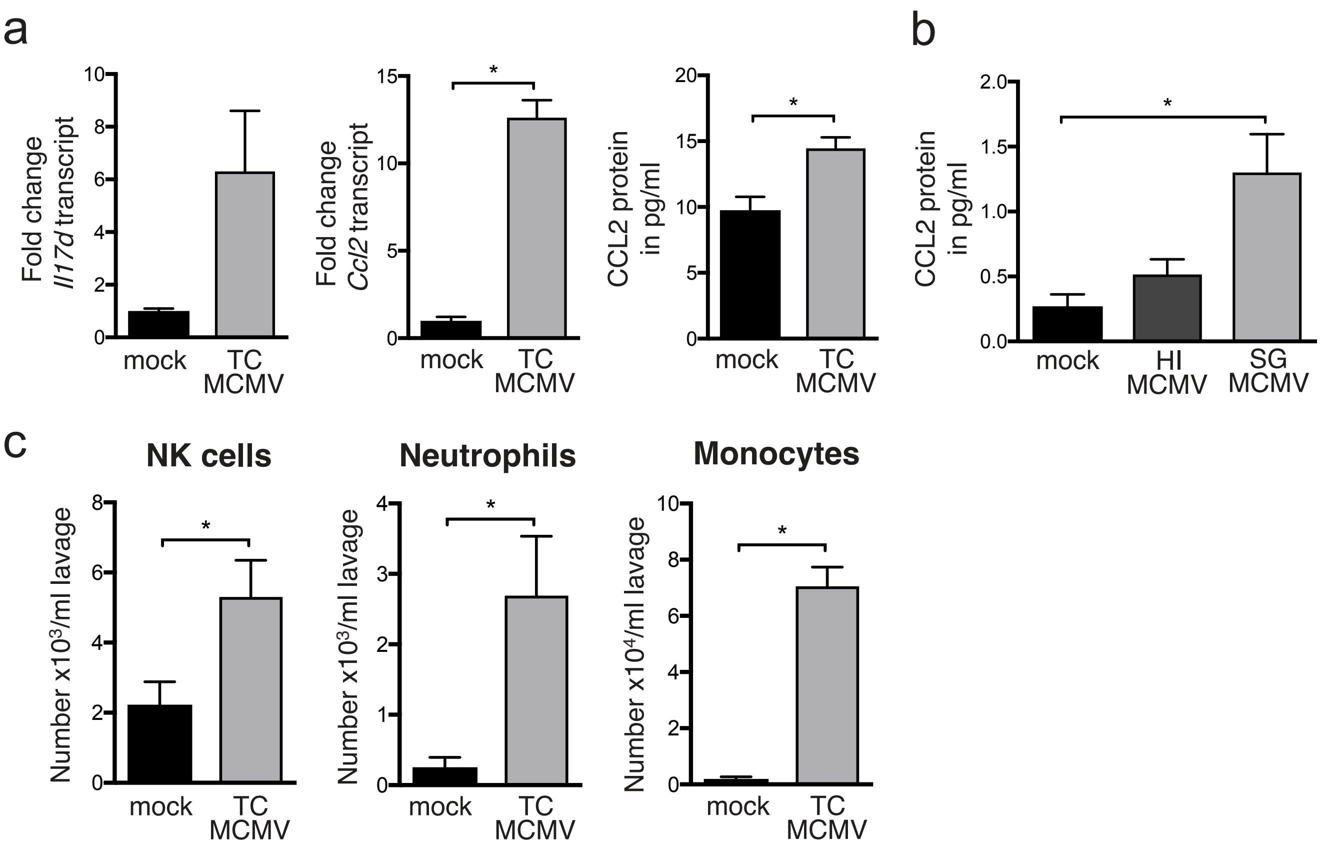

Mice were i.p. infected with $1x10^6$ pfu/mouse of tissue culture (TC)- derived (a,c) or $3x10^5$ pfu/mouse of salivary gland (SG)-derived (b) live or heat-inactivated (HI) MCMV, and *Il17d* and *Ccl2* transcript and CCL2 protein were measured by qPCR or ELISA after 24h. (c) Cells in the peritoneal lavage were counted and analyzed by FACS. Total numbers of NK cells, neutrophils and monocytes are shown. Data are represented as mean ± SEM.

Supplementary Figure S2. FACS gating strategy.

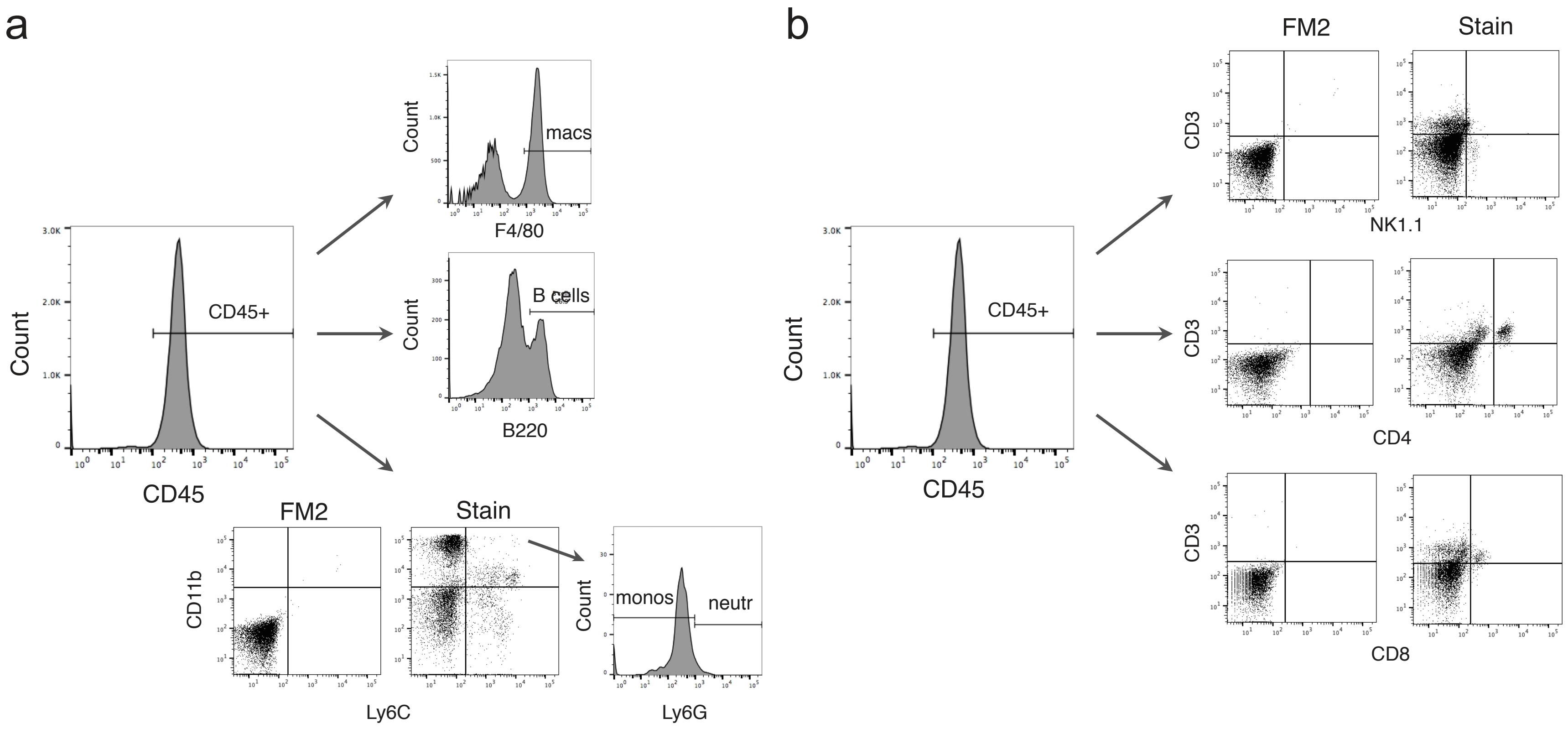

Representative FACS gating strategy of myeloid cells (a) and lymphocytes (b) in the peritoneum of a mock-infected WT mouse, gated on 7AAD$^-$ (live) cells. CD45$^+$ cells were determined as F4/80$^+$ (macs=macrophages), B220$^+$ (B cells), CD11b$^+$/Ly6C$^+$/Ly6G^{low} (monos= monocytes/macrophage precursors), CD11b$^+$/Ly6C$^+$/Ly6G^{high} (neutr=neutrophils), CD3$^-$/NK1.1$^+$ (NK cells), CD3$^+$/CD4$^+$ (CD4$^+$ T cells) and CD3$^+$/CD8$^+$ (CD8$^+$ T cells). FM2=Fluorescence minus two.

Supplementary Figure S3. Immune phenotyping of WT and *Il17d*$^{-/-}$ mice.

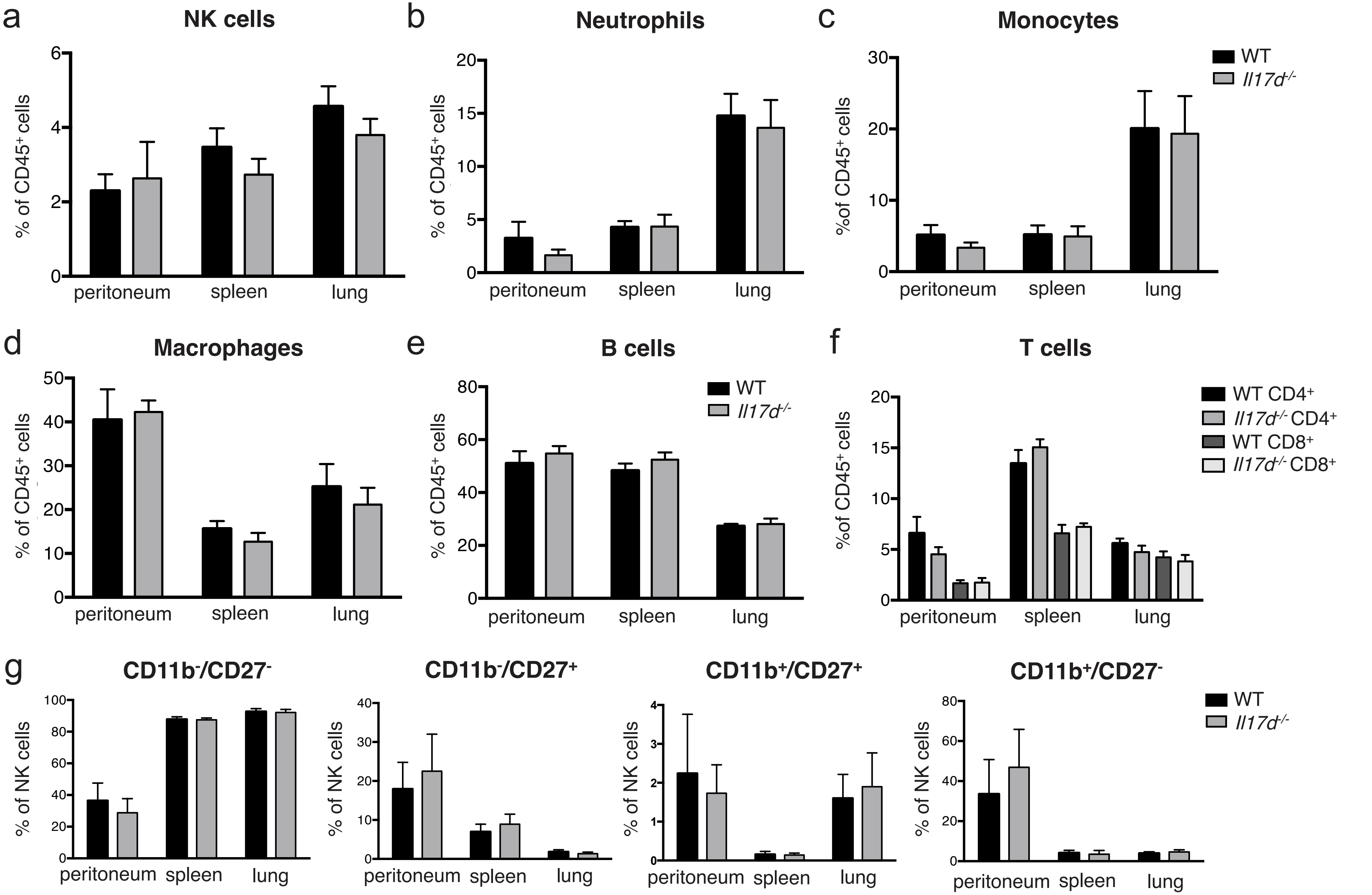

Shown are NK cells (a), neutrophils (b), monocytes (c), macrophages (d), B cells (e), T cells (f) and maturation status of NK cells based on CD11b/CD27 expression (g) in peritoneum, spleen and lung. Data are represented as mean ± SEM.

Supplementary Figure S4. Percentages of NK cells expressing Ly49H or CD69 after MCMV infection in the peritoneum, spleen and lung.

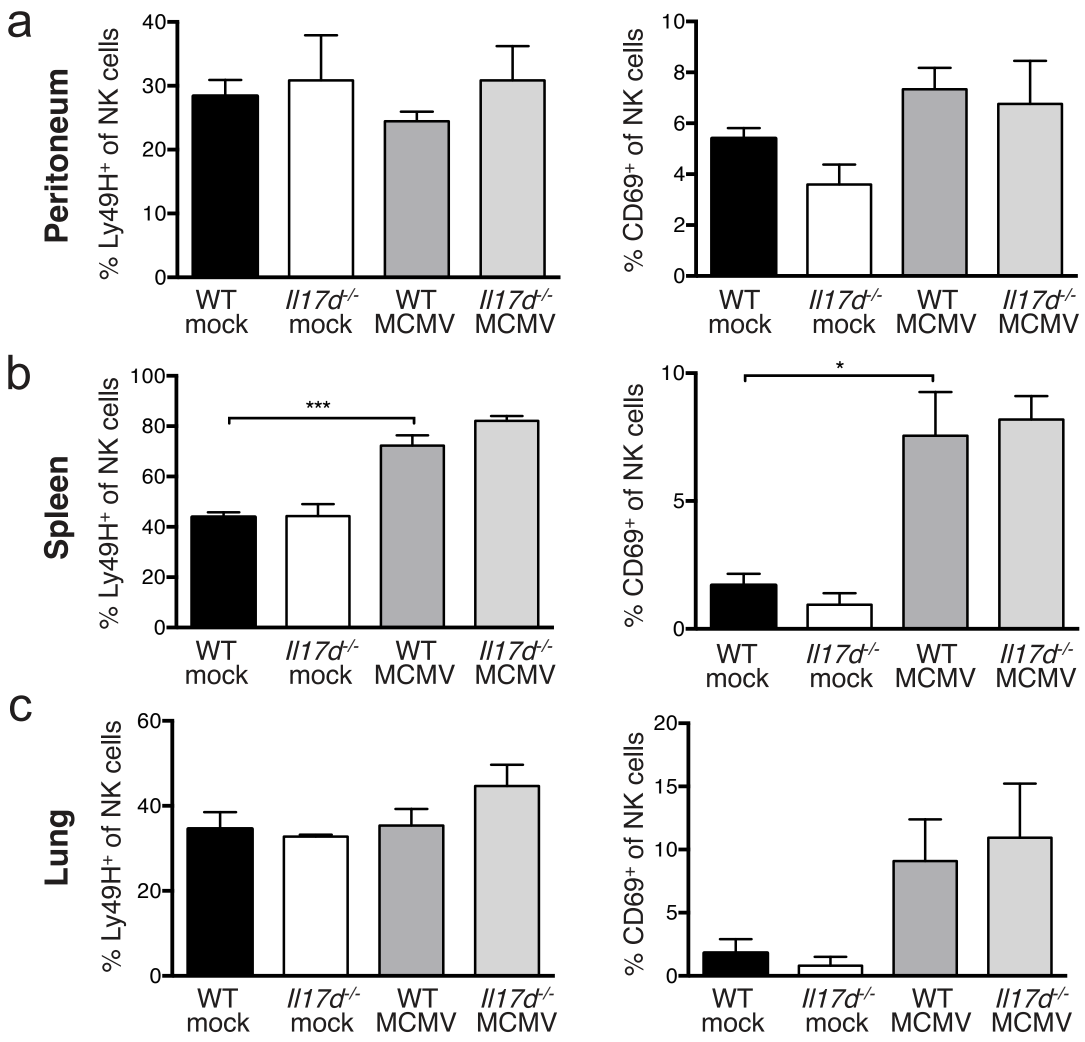

Mice were i.p. infected with $3x10^5$ pfu/mouse of MCMV (or mock) and cells in the peritoneum, spleen or lung were analyzed by FACS 24 hours (a) or 5 days (b,c) later. Percentage of NK cells expressing the surface receptor Ly49H (left) or CD69 (right) in WT or *Il17d*$^{-/-}$ mice. Data are represented as mean ± SEM.

Supplementary Figure S5. Immune cell recruitment into peripheral organs of MCMV-infected mice.

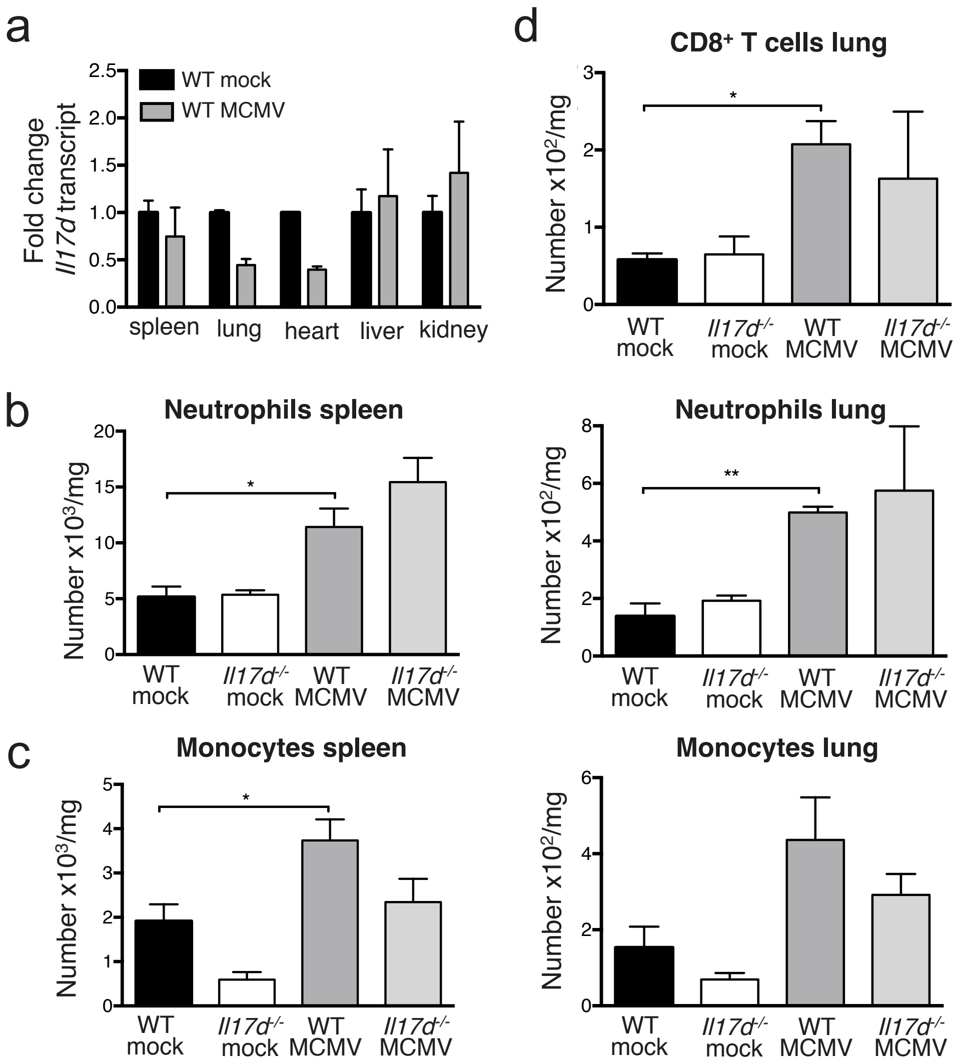

Mice were i.p. infected with $3x10^5$ pfu/mouse of MCMV (or mock) and peripheral organs were analyzed 5 days later.
(a) *Il17d* expression in spleen, lung, heart, liver and kidney.
(b)- (d) Total numbers of neutrophils (b), monocytes/macrophage precursors (c) and $CD8^+$ T cells (d) in spleen and lung of WT and *Il17d*$^{-/-}$ mice. Data are represented as mean ± SEM.

Supplementary Figure S6. Immune phenotyping of WT and *Nrf2*$^{-/-}$ mice.

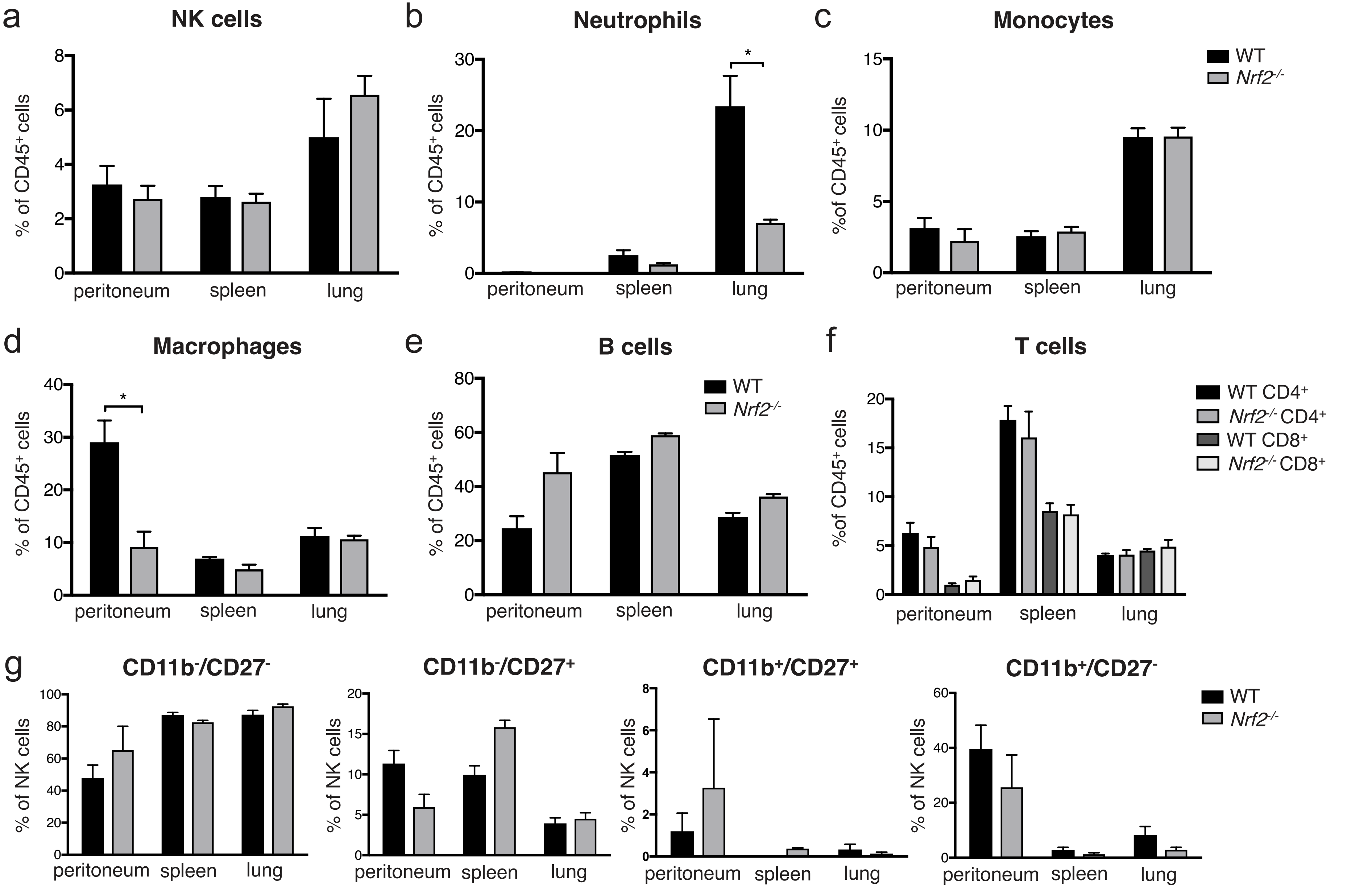

Shown are NK cells (a), neutrophils (b), monocytes (c), macrophages (d), B cells (e), T cells (f) and maturation status of NK cells based on CD11b/CD27 expression (g) in peritoneum, spleen and lung. Data are represented as mean ± SEM.
